# Analysis of Parkinson's disease brain–derived DNA for alpha-synuclein coding somatic mutations

**DOI:** 10.1002/mds.25883

**Published:** 2014-04-21

**Authors:** Christos Proukakis, Maryiam Shoaee, James Morris, Timothy Brier, Eleanna Kara, Una-Marie Sheerin, Gavin Charlesworth, Eduardo Tolosa, Henry Houlden, Nicholas W Wood, Anthony H Schapira

**Affiliations:** 1 Department of Clinical Neuroscience, Institute of Neurology, University College LondonLondon, United Kingdom; 2 Department of Molecular Neuroscience, Institute of Neurology, University CollegeLondon, London, UK; 3 Neurology Service, Hospital Clínic de Barcelona, Universitat de Barcelona, IDIBAPS, Centro de Investigación Biomédica en Red sobre Enfermedades Neurodegenerativas (CIBERNED)Barcelona, Catalonia, Spain

**Keywords:** SNCA, alpha-synuclein, somatic mutation, mosaicism, etiology of Parkinson's disease

## Abstract

**Background:**

Although alpha-synuclein (SNCA) is crucial to the pathogenesis of Parkinson's disease (PD) and dementia with Lewy bodies (DLB), mutations in the gene appear to be rare. We have recently hypothesized that somatic mutations in early development could contribute to PD.

**Methods:**

Expanding on our recent negative small study, we used high-resolution melting (HRM) analysis to screen *SNCA* coding exons for somatic point mutations in DNA from 539 PD and DLB cerebellar samples, with two additional regions (frontal cortex, substantia nigra) for 20 PD cases. We used artificial mosaics to determine sensitivity where possible.

**Results:**

We did not detect any evidence of somatic coding mutations. Three cases were heterozygous for known silent polymorphisms. The protocol we used was sensitive enough to detect 5% to 10% mutant DNA.

**Conclusion:**

Using DNA predominantly from cerebellum, but also from frontal cortex and substantia nigra (n = 20 each), we have not detected any somatic coding *SNCA* point mutations. © 2014 The Authors. Movement Disorders published by Wiley Periodicals, Inc. on behalf of International Parkinson and Movement Disorder Society.

Alpha-synuclein (SNCA), the major component of Lewy bodies, has a key role in the pathogenesis of Parkinson's disease (PD) and dementia with Lewy bodies (DLB). Occasional copy number variants (CNV, duplications and triplications), and five pathogenic point mutations or single nucleotide variants (SNVs), have been reported in PD, usually in familial cases.^1^ Noncoding *SNCA* variation is a risk factor for sporadic PD.^2^ The known inherited genetic risk factors may still account for only 10% to 20% of the total PD risk,^3^ and the initiating event in SNCA aggregation is still debated. We have recently proposed in this journal that post-zygotic somatic mutations in *SNCA*, occurring in early embryogenesis, could contribute to PD, although our pilot study was negative.^4^ Somatic mutations can lead to the presence of more than one genetically distinct cell in a single organism (mosaicism)^5^ and could be absent in peripheral lymphocyte DNA, which is usually analyzed. Somatic mutations have been suggested as a cause of sporadic neurodegenerative disorders^6^,^7^ and as an explanation of nonheritable disease in general.^8^ Here we expand on our recent study,^4^ investigating the hypothesis of somatic mutations by searching for evidence of low-level coding *SNCA* point mutations in a large series of PD and DLB brain DNA.

## Materials and Methods

### Samples Used

DNA from brains of 511 cases with idiopathic PD and 28 with DLB was analyzed. Patients had given informed consent for use of their brains in research, and the study was approved by the local ethics committee. The brain samples originated from the Queen Square brain bank, UK (PD, n = 339), the Parkinson's UK Tissue bank (PD, n = 105), and the Neurological Tissue Bank of the Biobanc-Hospital Clinic-Institut d'Investigacions Biomèdiques August Pi i Sunyer, Spain (n = 95, of which 67 were PD and 28 were DLB). DNA was extracted from the cerebellum in all cases, using a Qiagen Blood and Tissue kit, and additionally from the substantia nigra (SN) and frontal cortex in 20 PD cases. The PD cases for study of all three brain regions were chosen from the Parkinson's UK brain bank based on the combination of relatively short disease duration (mean, 7.7 ± 3 years; considered more likely to have surviving neurons with mutations in SN^4^) and, where possible, relatively early age of onset (mean, 64.1 ± 6 years; considered more likely to have somatic mutations^6^).

### Laboratory Methods

We analyzed all samples using high resolution melting curve (HRM) analysis of *SNCA* coding exons (numbers 2-6 for ENSEMBL transcript ID 394986) with previously reported primers^4^; this allows detection of low-level mosaicism caused by somatic mutations (SNV and small insertions/deletions), which could be missed by Sanger sequencing, which is less sensitive.^9^ Details are provided in Supplementary Data online. To determine the lowest level at which a somatic mutation would be detectable, we created artificial mosaics by diluting DNA carrying a heterozygous SNV with wild-type DNA. Dilutions down to 1:20 (i.e., 5% of DNA with heterozygous SNV, or 2.5% mutant DNA level) were studied in triplicate.

## Results

**Figure 1:**
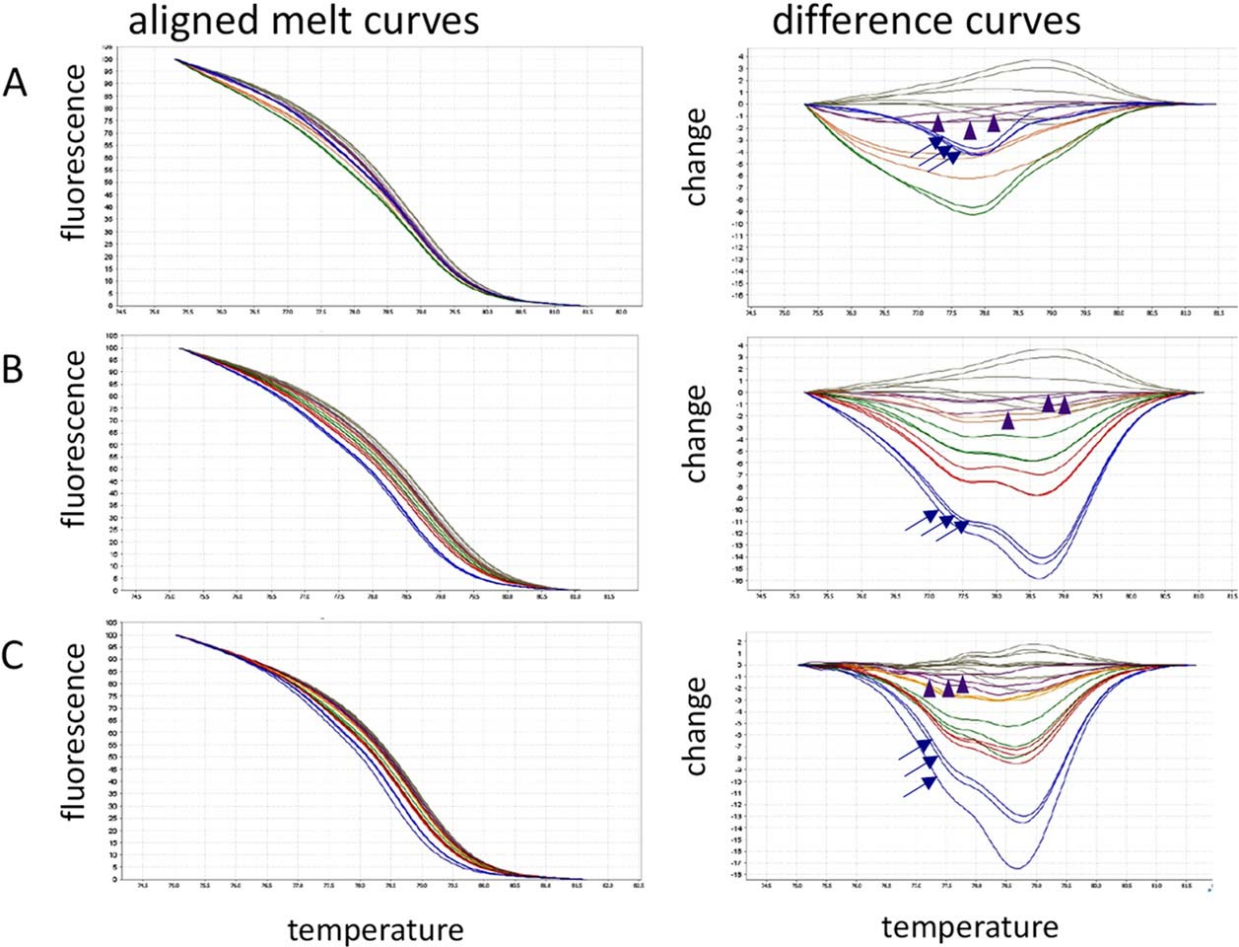
Estimation of sensitivity of HRM for detection of low levels of exon 3 SNV. A: H50Q, B: A53T, C: G51D. The undiluted heterozygous sample (50% mutation) is blue and identified by arrows, and lower levels of mutations are 20% (red), 10% (green), 5% (orange), 2.5% (purple, identified by arrowheads). Controls are shown in gray. [Color figure can be viewed in the online issue, which is available at http://wileyonlinelibrary.com.]

We first established the sensitivity of our HRM analysis by determining the lowest levels at which known pathogenic exon 3 mutations (H50Q, A53T) could be differentiated from control DNA (Figure 1a, b). The HRM analysis showed that mutant DNA proportions of at least 5% (equivalent to 10% of cells from a sample carrying a heterozygous somatic SNV) were differentiated from controls. Detection of the G51D mutation^10^ allowed us to subsequently confirm that this could be differentiated at a level of 5% (Figure 1c). In all of these cases, even a 2.5% level of mutation gave a minimal difference, but this probably would have been detected only in H50Q. Sequencing of artificial mosaics for selected SNVs confirmed the superiority of HRM over sequencing for low-level mutations (Supplemental Data Figure 1).

**Figure 2:**
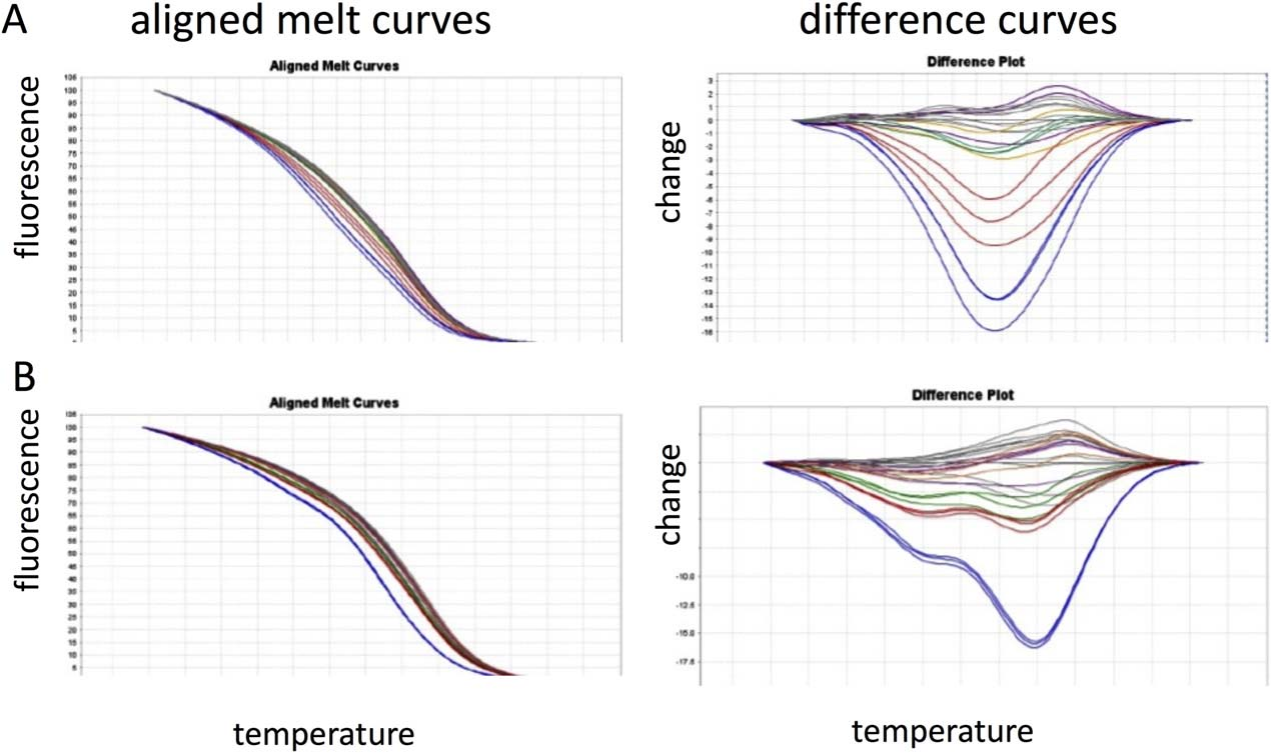
Estimation of sensitivity of HRM for detection of low levels of SNV in other exons. A: rs144758871 (exon 4), B: rs76642636 (exon 6). The undiluted heterozygous sample (50% SNV) is blue, and SNV levels in diluted samples are 20% (red), 10% (green), 5% (orange), 2.5% (purple). Controls are shown in gray. [Color figure can be viewed in the online issue, which is available at http://wileyonlinelibrary.com.]

Coding exons were amplified and analyzed by HRM in all samples in duplicate. Samples in which at least one of the replicates showed an aberrant melt curve were analyzed with HRM in additional duplicate or more reactions, and a polymerase chain reaction product with the apparent shift was sequenced bidirectionally. Only three samples showed clear melting curve shifts; sequencing of these did not reveal somatic mutations, but heterozygosity for synonymous silent polymorphic SNVs already present in dbSNP; rs76642636 (exon 6, c.324C>T) in two cases, and rs144758871 (exon 4, c.216G>A) in one (Figure 2). Although even silent SNVs could have biologically important effects,^11^ the very low frequency of these SNVs, both in our patient cohort and in the population (0.4% and 0.07%, in European and African American controls, respectively, in the Washington Exome server database, http://evs.gs.washington.edu/EVS/), does not allow any conclusions on whether they might modulate PD risk. Having detected these SNVs, however, we then used them to create artificial mosaics to determine the sensitivity of HRM for a low percentage of these changes too; detection of 10% variant DNA level was possible, but lower levels could not be reliably differentiated (Figure 2).

No additional consistent melting curve shifts were detected. We considered the possibility that subtle HRM shifts could indicate mutations at levels below Sanger sequencing sensitivity. However, in no cases did a sample give a consistently aberrant profile for a particular exon, which would have suggested that possibility.

## Discussion

We report the largest series of PD and DLB brain-derived DNA analyzed for somatic *SNCA* coding mutations. We studied 539 DNA samples from cerebellum, and in 20 PD cases, also SN and frontal cortex, using HRM, which has proven sensitivity for low-level somatic mutations. No somatic mutations were identified. Known heterozygous silent SNVs, not leading to amino acid changes, accounted for the only consistent HRM abnormalities. We demonstrated that several SNVs could be confidently detected by our protocol at mutation level 5% or lower in exon 3 (where 4 of 5 missense mutations are located), and 10% for exons 4 and 6, in line with the known sensitivity of HRM; we expect to have achieved similar sensitivity for other exons, although we could not test that because of lack of positive controls. We can therefore confidently exclude a moderate level of mosaicism (10% level of mutant DNA, or 20% cells with a heterozygous mutation, and even lower for exon 3) for *SNCA* coding mutations (SNVs and small insertions or deletions) in the brain regions analyzed.

Our technique was not designed to detect even lower level coding somatic mutations, which could still be important, however, because they could act as the seed from which pathological conditions spread in a prion-like fashion,^3^ noncoding mutations, or CNVs. The possibility of somatic mutations in other regions could not be excluded, because sampling bias could underlie negative results,^3^ although for 20 cases we analyzed three regions, including the SN. The SN may be the most relevant region, because it is very heavily affected in PD, and therefore any somatic mutations should be present there^3^; conversely, however, SN neurons with somatic mutations should be the most vulnerable, and therefore could be lost long before the patient dies. The cell lineage of the brain is still very poorly understood,^3^ but most brain cell types show little clonal clustering, suggesting that cells with somatic mutations in early development could be widely dispersed and present at low levels in any given region^12^; therefore the cerebellum, where neurons are not significantly lost in PD, although some SNCA accumulation is present,^13^ could also allow detection of putative very early somatic mutations present in neurons of a shared lineage dispersed throughout the nervous system.

The results of this large study, in conjunction with our recent smaller study, provide evidence against somatic *SNCA* coding SNVs (at levels of at least 5-10%) being a major cause of PD. Although advances in next-generation sequencing allow the detection of ever-decreasing levels of SNVs, we believe that further efforts to detect somatic mutations in PD should focus on other kinds of genomic changes. The de novo mutation rate for CNV can be two to four orders of magnitude higher than for SNV,^14^ with CNVs arising in mitosis, explaining the increasing evidence for CNV mosaicism in healthy humans, including the brain.^15^–^17^ This was elegantly demonstrated in single frontal cortical neurons, which frequently harbor somatic CNVs.^18^,^19^ Both *SNCA* and *PARK2* may be particularly susceptible to somatic CNV generation, because they are in chromosomal fragile sites.^4^,^20^ The concept of widespread mosaicism is gaining ground,^8^,^21^,^22^ and a substantial proportion of the risk of sporadic diseases may be explained by nonheritable somatic genomic variation, possibly in genes involved in Mendelian forms of the same disease.^8^ Another common sporadic disease with complex genetics, hypertension can be attributable to somatic mutations in genes involved in aldosterone production, with a more severe phenotype in inherited cases.^23^–^25^ Because most PD risk remains unexplained, further analysis for somatic mutations could advance our understanding of sporadic PD pathogenesis, but evidence has yet to be provided to support this hypothesis, and we must acknowledge the potential that this concept is not relevant to PD.

## References

[b1] Kasten M, Klein C (2013). The many faces of alpha-synuclein mutations. Mov Disord.

[b2] Nalls MA, Plagnol V, Hernandez DG (2011). Imputation of sequence variants for identification of genetic risks for Parkinson's disease: a meta-analysis of genome-wide association studies. Lancet.

[b3] Engeholm M, Gasser T (2013). Parkinson's disease: is it all in the Genes?. Mov Disord.

[b4] Proukakis C, Houlden H, Schapira AH (2013). Somatic alpha-synuclein mutations in Parkinson's disease: hypothesis and preliminary data. Mov Disord.

[b5] Biesecker LG, Spinner NB (2013). A genomic view of mosaicism and human disease. Nat Rev Genet.

[b6] Frank SA (2010). Somatic evolutionary genomics: mutations during development cause highly variable genetic mosaici'm with risk of cancer and neurodegeneration. Proc Natl Acad Sci U S A.

[b7] Pamphlett R (2004). Somatic mutation: a cause of sporadic neurodegenerative diseases?. Med Hypotheses.

[b8] Forsberg LA, Absher D, Dumanski JP (2013). Non-heritable genetics of human disease: spotlight on post-zygotic genetic variation acquired during lifetime. J Med Genet.

[b9] Wittwer CT (2009). High-resolution DNA melting analysis: advancements and limitations. Hum Mutat.

[b10] Kiely A, Asi YT, Kara E (2013). Alpha-synucleinopathy associated with G51D SNCA mutation: a link between Parkinson's disease and multiple system atrophy?. Acta Neuropathol.

[b11] Korvatska O, Strand NS, Berndt JD (2013). Altered splicing of ATP6AP2 causes X-linked parkinsonism with spasticity (XPDS). Hum Mol Genet.

[b12] Poduri A, Evrony GD, Cai X, Walsh CA (2013). Somatic mutation, genomic variation, and neurological disease. Science.

[b13] Mori F, Piao YS, Hayashi S (2003). Alpha-synuclein accumulates in Purkinje cells in Lewy body disease but not in multiple system atrophy. J Neuropathol Exp Neurol.

[b14] Lupski JR (2012). Brain copy number variants and neuropsychiatric traits. Biol Psychiatry.

[b15] Abyzov A, Mariani J, Palejev D (2012). Somatic copy number mosaicism in human skin revealed by induced pluripotent stem cells. Nature.

[b16] Mkrtchyan H, Gross M, Hinreiner S (2010). Early embryonic chromosome instability results in stable mosaic pattern in human tissues. PLoS One.

[b17] O'Huallachain M, Karczewski KJ, Weissman SM, Urban AE, Snyder MP (2012). Extensive genetic variation in somatic human tissues. Proc Natl Acad Sci U S A.

[b18] McConnell MJ, Lindberg MR, Brennand KJ (2013). Mosaic copy number variation in human neurons. Science.

[b19] Gole J, Gore A, Richards A (2013). Massively parallel polymerase cloning and genome sequencing of single cells using nanoliter microwells. Nat Biotechnol.

[b20] Fungtammasan A, Walsh E, Chiaromonte F, Eckert KA, Makova KD (2012). A genome-wide analysis of common fragile sites: what features determine chromosomal instability in the human genome?. Genome Res.

[b21] Macosko EZ, McCarroll SA (2013). Genetics. Our fallen genomes. Science.

[b22] Lupski JR (2013). Genetics: genome mosaicism—one human, multiple genomes. Science.

[b23] Azizan E, Poulsen H, Tuluc P (2013). Somatic mutations in ATP1A1 and CACNA1D underlie a common subtype of adrenal hypertension. Nat Genet.

[b24] Beuschlein F, Boulkroun S, Osswald A (2013). Somatic mutations in ATP1A1 and ATP2B3 lead to aldosterone-producing adenomas and secondary hypertension. Nat Genet.

[b25] Scholl UI, Goh G, Stolting G (2013). Somatic and germline CACNA1D calcium channel mutations in aldosterone-producing adenomas and primary aldosteronism. Nat Genet.

