# Supplementary figures and images for: Analysis of Parkinson's disease brain–derived DNA for alpha-synuclein coding somatic mutations
Source: Mov Disord. 2014 Apr 21;29(8):1060–4. doi: 10.1002/mds.25883 (PMC4190821; doi:10.1002/mds.25883)

## Slide 1
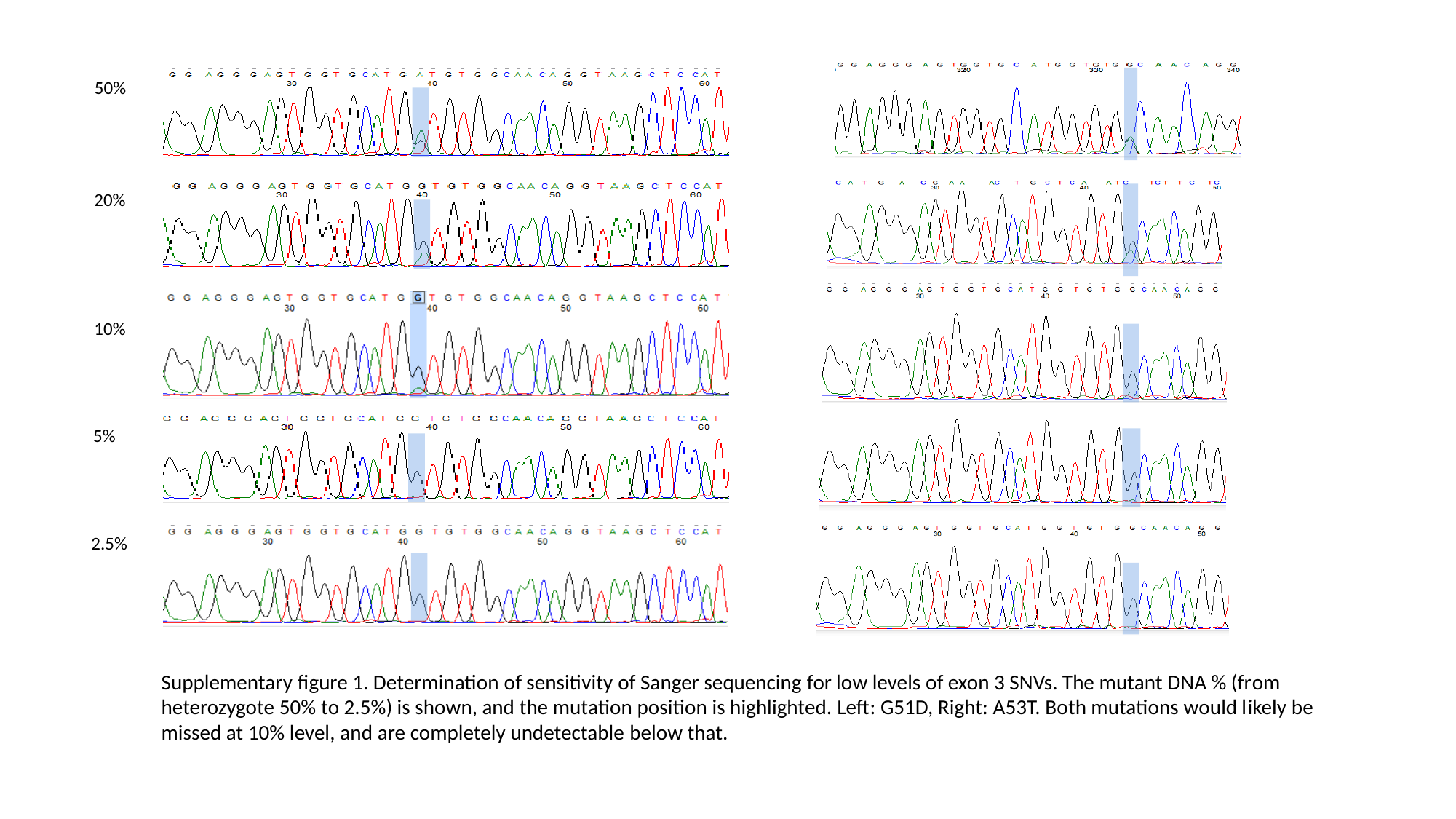

Supplement: Supplementary file 1 [file mds0029-1060-sd1.pptx]
