# Supplementary material for: Analysis of Parkinson's disease brain–derived DNA for alpha-synuclein coding somatic mutations
Source: Mov Disord. 2014 Apr 21;29(8):1060–4. doi: 10.1002/mds.25883 (PMC4190821; doi:10.1002/mds.25883)
Supplement: Supplementary file 2 [file mds0029-1060-sd2.docx]

| **Exon (amplicon size in bp)** | **Primer volume per reaction**  (μl of each, from 10μM stock) | **Temperature (°C)** |
| --- | --- | --- |
| **2 (187)** | 0.3 | 60 |
| **3 (107)** | 0.48 | 58 |
| **4 (209)** | 0.22 | 60 |
| **5 (160)** | 0.6 | 61 |
| **6 (97)** | 0.6 | 59 |

Supplementary table 1. PCR conditions. A combined annealing and extension temperature was used for 60 seconds, as suggested by the manufacturer. The initial denaturation step was 95°C for 10 minutes, with a 15 second denaturation in each cycle.
