# Supplementary material for: Analysis of Parkinson's disease brain–derived DNA for alpha-synuclein coding somatic mutations
Source: Mov Disord. 2014 Apr 21;29(8):1060–4. doi: 10.1002/mds.25883 (PMC4190821; doi:10.1002/mds.25883)
Supplement: Supplementary file 3 [file mds0029-1060-sd3.docx]

We used the Lifetech StepOne, which allows PCR amplification followed by HRM analysis as a single experiment in the same machine, simplifying the workflow from our previous method of transferring PCR products for melting on a separate machine (the Idaho Systems Lightscanner). [^4^](#_ENREF_4) PCR primer concentration and temperature were individually optimised for this system for each exon (supplementary table 1). We performed a 40-cycle amplification using 20 ng DNA template in a 20 μl reaction volume with MeltDoctor mix (Lifetech), with a combined annealing / extension step according to the manufacturer protocol, and subsequently denatured DNA at 95° C for 10 seconds, allowed reannealing at 60° C for 1 minute, and then recorded fluorescence during melting to 95° C. All reactions were initially performed in duplicate, and analysed using High Resolution Melting software v3.0 (Lifetech). Amplicons which gave curves which appeared aberrant, either as determined automatically by the software, or by visual inspection by two researchers, were selected for further analysis. Where sequencing was required, amplicons were purified using Qiagen spin columns and sequenced bidirectionally using the HRM primers at the UCL core sequencing facility; all electopherograms were carefully inspected visually for evidence of low peaks which could signify low level mosaic mutations. [^10^](#_ENREF_10) As HRM platforms may differ in their sensitivity, [^11^](#_ENREF_11) and we are not aware of any direct comparisons between the two systems we have used, we also sought to determine the sensitivity for low levels of SNV, including those we had previously analysed on the Lightscanner. [^4^](#_ENREF_4)
